# RAI14 Promotes Melanoma Progression by Regulating the FBXO32/c-MYC Pathway

**DOI:** 10.3390/ijms231912036

**Published:** 2022-10-10

**Authors:** Jie Xu, Pengfei Shi, Fanwei Xia, Xuan Zhao, Junfan Chen, Rui Geng, Hongjuan Cui, Liqun Yang

**Affiliations:** 1 State Key Laboratory of Silkworm Genome Biology, Institute of Sericulture and Systems Biology, Southwest University, Chongqing 400716, China; 2 Cancer Center, Medical Research Institute, Southwest University, Chongqing 400716, China

**Keywords:** RAI14, c-MYC, ubiquitination, melanoma, proliferation

## Abstract

Melanoma originates from the malignant transformation of melanocytes. Compared with other skin cancers, melanoma has a higher fatality rate. The 5-year survival rate of patients with early-stage primary melanoma through surgical resection can reach more than 90%. However, the 5-year survival rate of patients with metastatic melanoma is only 25%. Therefore, accurate assessment of melanoma progression is critical. Previous studies have found that Retinoic Acid Induced 14(RAI14) is critical in tumorigenesis. However, the biological function of RAI14 for the development of melanoma is unclear. In this study, RAI14 is highly expressed in melanoma and correlated with prognosis. The expression of RAI14 can affect the proliferation, migration and invasion of melanoma cells. F-Box Protein 32(FBXO32) is an E3 ubiquitin ligase of c-MYC. We found that RAI14 affects the transcriptional expression of FBXO32 and regulates the stability of c-MYC. These results suggest that RAI14 play an important role in the growth of melanoma and is expected to be a therapeutic target for melanoma.

## 1. Introduction

Melanoma skin cancer is derived from skin melanocytes and has a high risk of metastatic spread. Melanoma mortality accounts for 90% of all skin cancer [1]. The incidence of melanoma is growing faster than any other preventable cancer currently [2]. Identifying the progression of melanoma is critical because it is “curable” in its early stages. However, it is difficult to treat when melanoma has metastasized. The 5-year survival rate of patients with metastatic melanoma is only 25% [3]. At present, melanoma is mainly treated by surgical resection, immunotherapy, gene therapy and chemotherapy [4]. In the past decade, although researchers have developed some new drugs for metastatic melanoma, most patients have developed drug resistance. Therefore, there is an urgent need to develop molecular biomarkers for the early identification of high-risk melanoma.

*RAI14*, also known as *NORPEG* or *Ankycorbin*, is a gene induced by all-trans retinoic acid first discovered in human retinal pigment epithelial cells, located on human chromosome 5p13.2~13.3. RAI14 protein contains six ankyrin repeats and two coiled-coil domains [5]. Recently, more and more studies have shown that ankyrin repeat domain-containing ankyrins are involved in the biological functions of eukaryotic cells, such as tumorigenesis (Ink4, 53BP2) [6], transcriptional regulation (IKB, Mbp1, RFXANK) [7], signal transduction and regulation of inflammatory responses, etc. [8,9]. RAI14 is associated with super-enhancers and is a new potential biomarker for lung adenocarcinoma [10]. Many recent studies have explored the mechanism of RAI14 on tumor growth [11,12,13]. In addition, RAI14 is associated with tumor chemosensitivity [14]. However, the role of RAI14 in melanoma development remains unclear.

*c-MYC* is an oncogene that can affect tumor cell proliferation, migration, invasion, metabolism, ribosome production, etc. [15,16,17]. FBXO32 (MAFbx/Atrogin-1), an E3 ubiquitin ligase, has been reported to target the oncoprotein c-MYC for ubiquitination and degradation through the proteasome pathway [18]. In this study, we found that the expression of RAI14 affects the proliferation, migration and invasion of melanoma cells. At the same time, we found that the downstream target of RAI14 is FBXO32, which is involved in the ubiquitination of c-MYC. In other words, RAI14 could inhibit the expression of FBXO32 to regulate the stability of c-MYC. In summary, our data reveal an important role of RAI14 in melanoma development. It also indicated that RAI14 may be a potential target for the treatment of melanoma.

## 2. Results

### 2.1. High Expression of RAI14 Is Associated with Poor Prognosis of Melanoma 

**Figure 1:**
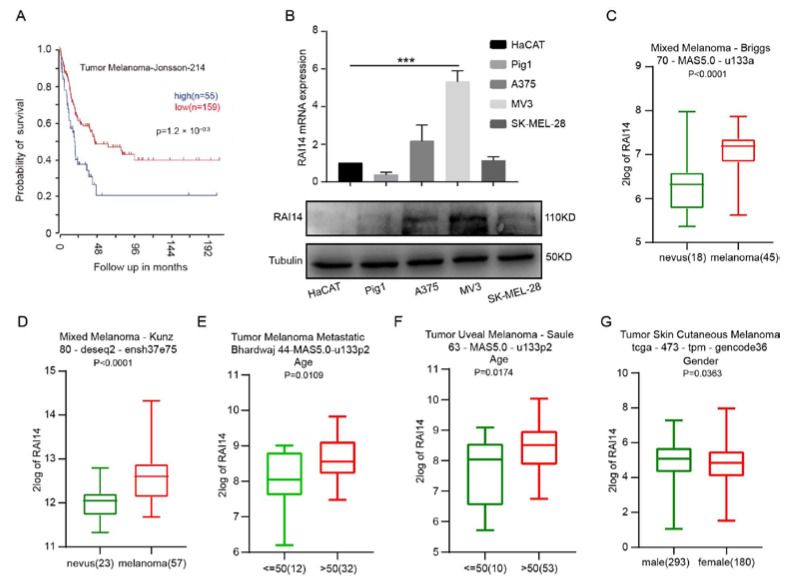
High expression of RAI14 is associated with poor prognosis of melanoma. (**A**) Result of the Kaplan–Meier analysis of progression-free survival and the log-rank test *p* values are from Tumor Melanoma-Jonsson-214 in R2 database. (**B**) qRT-PCR and Western blot were used to examine expression of RAI14 in melanoma cell lines (A375, MV3 and SK-MEL-28), normal human epidermal melanocytes (Pig1) and human immortalized epidermal cells (HaCAT). (**C**,**D**) Comparison of RAI14 expression levels in nevus and melanoma by database. (**E**–**G**) The relationship between RAI14 expression and patient’s age and gender. The data were expressed as mean ± SD. Student’s *t* test and One-way ANOVA were performed to analyze significance. *** *p* < 0.001.

We first explored the correlation between RAI14 and the prognosis of melanoma patients. We analyzed the survival data from the R2 database and found that RAI14 high expression was associated with the poor prognosis of melanoma patients (Tumor Melanoma-Jonsson-214). Melanoma patients with high RAI14 expression have a shorter survival time than patients with low RAI14 expression (Figure 1A). Then, we analyzed the expression of RAI14 in melanoma cell lines (A375, MV3 and SK-MEL-28), normal human epidermal melanocytes (Pig1) and immortalized human epidermal cells (HaCAT) by RT-PCR and Western blotting. We found that the expression of RAI14 was higher in MV3 than in other cell lines (Figure 1B). We then found that the expression of RAI14 was significantly higher in melanoma than in nevus by analyzing two databases (Figure 1C,D). Subsequently, the expression of RAI14 was also correlated with the age and sex of the patients through analyzing the database (Figure 1E–G). The expression of RAI14 was higher in dead patients than in living patients according to four different data sets (Figure S1A–D). The expression level of RAI14 in with-tumor melanoma patients was significantly higher than tumor-free melanoma patients (Figure S1E,F). Moreover, the expression of RAI14 in the Pten wild-type status is significantly lower than in the Pten mutation status (Figure S1G). We also explored the subcellular localization of RAI14 in melanoma cells. Immunofluorescence experiments showed that RAI14 was expressed in both nucleus and cytoplasm (Figure S2H). In summary, the above results indicate that high RAI14 expression was associated with a poor prognosis of melanoma patients.

### 2.2. RAI14 Knockdown Inhibited the Cell Proliferation, Migration and Invasion of Melanoma Cells 

**Figure 2:**
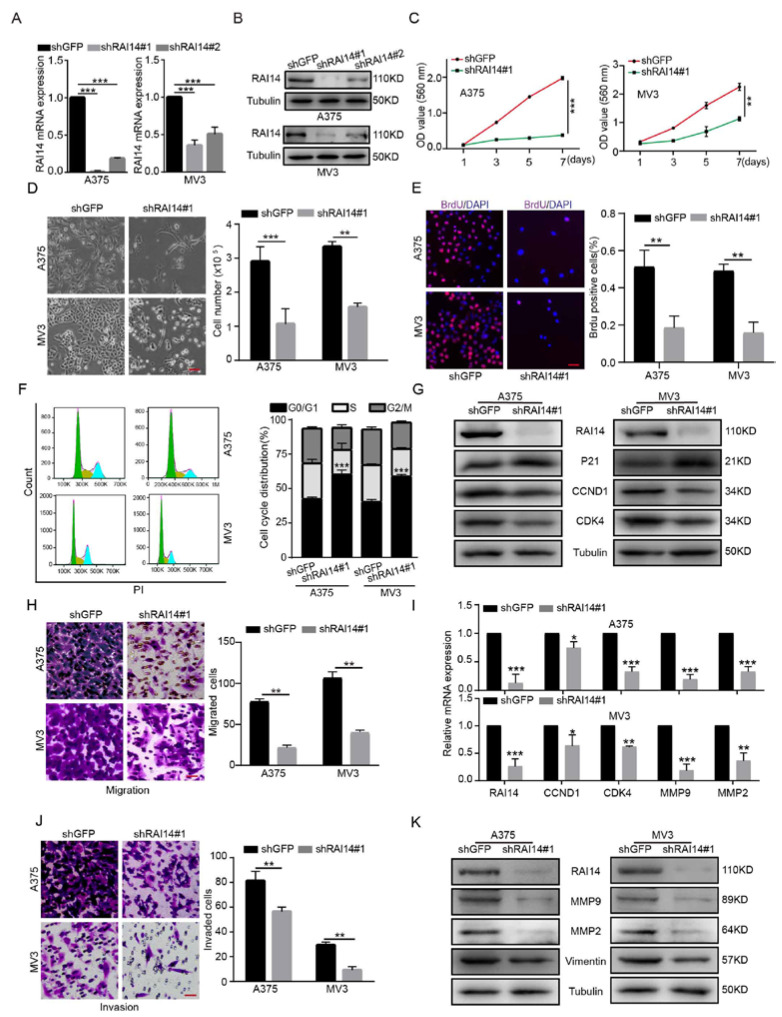
RAI14 knockdown inhibited the cell proliferation, migration and invasion of melanoma cells. (**A**,**B**) The knockdown efficiency of RAI14 was detected by Western blot and qRT-PCR. (**C**) Cell viability was detected by MTT assay after knockdown of RAI14. (**D**) Microscopic observation and cell-counting assays were used to detect the proliferation of melanoma cells. Scale bar = 50 μm. (**E**) DNA synthesis capacity was examined by BrdU assay after RAI14 was knocked down. Scale bar = 50 μm. (**F**) Flow cytometry assays were performed to detect the effect of RAI14-knockdown on the cell cycle. (**G**) The expression of G1 cell-cycle regulatory protein was detected after RAI14 knockdown. (**H**,**J**) Transwell assay was performed to detect the effect of RAI14 knockdown on cell migration and invasion. Scale bar = 50 μm. (**I**) The effects of RAI14 knockdown on the mRNA expression levels of cell-cycle-related genes and EMT-related genes were investigated by qPCR experiments. (**K**) EMT related-proteins were detected in RAI14 knockdown cells and control cells. The data were expressed as mean ± SD. Student’s *t* test and One-way ANOVA were performed to analyze significance. * *p* < 0.05, ** *p* < 0.01, *** *p* < 0.001.

To explore the role of RAI14 in melanoma cells, we constructed two shRNA sequences using siRNA technology: shRAI14#1 and shRAI14#2. It was verified that shRAI14 #1 had a better knockdown effect on RAI14 by RT-PCR and Western blotting assay (Figure 2A,B). After RAI14 was knocked down, the number of cells decreased and the cell morphology changed significantly (Figure 2D). MTT assays demonstrated that the silencing of RAI14 significantly inhibited cell proliferation of melanoma cells (Figure 2C). We further performed the bromodeoxyuridine (BrdU), and the results indicated that knockdown of RAI14 significantly reduced DNA synthesis ability (Figure 2E and Figure S2). Then, flow cytometry analysis indicated that RAI14 downregulation induced G1 arrest in melanoma cells (Figure 2F). To further confirm the changes, we detected the expression levels of CCND1, CDK4 and P21 protein. The results suggested that the protein expressions of CCND1 and CDK4 were downregulated and P21 was increased (Figure 2G). We also found that the mRNA expression levels of *CCND1* and *CDK4* decreased after knockdown of RAI14 (F2I). Melanoma cells have a strong ability to migrate and invade. Therefore, we used transwell and matrigel to explore the effect of RAI14 on melanoma cell migration and invasion. We found that the knockdown of RAI14 significantly reduced the number of invasive and migrating cells (Figure 2H,J). We further found that the protein expressions of Vimentin, MMP2 and MMP9 were downregulated in RAI14 knockdown cells. (Figure 2K). The mRNA expression levels of *MMP9* and *MMP2* were decreased after knockdown of RAI14 (Figure 2I). In short, these results indicated that RAI14 knockdown suppressed cell proliferation, migration and invasion of melanoma cells in vitro. 

### 2.3. RAI14 Recovery Restored the Cell Proliferation and Migration of RAI14 Knockdown Melanoma Cells

To further verify the effect of RAI14 on the proliferation and migration of melanoma cells, we constructed the RAI14 overexpression vector. To explore the effect of overexpression of RAI14 on the proliferation and migration of melanoma cells in RAI14 knockdown cells, we performed an MTT assay, plate clone assay, transwell assay and WB assay. The WB assay result showed that overexpression of RAI14 in knockdown melanoma cell lines partially restored the expression of CDK4 and CCND1 (Figure S2A). MTT assays and plate clone assays showed that overexpression of RAI14 in knockdown melanoma cell lines restores cell proliferation (Figure S2B,C). At the same time, transwell assays demonstrated that overexpression of RAI14 could rescue cell migration in RAI14 knockdown cells (Figure S2D). Taken together, this indicates that RAI14 was essential for proliferation, migration and invasion of melanoma cells. 

### 2.4. RAI14 Regulates the Stability of c-MYC by Regulating c-MYC Ubiquitination

**Figure 3:**
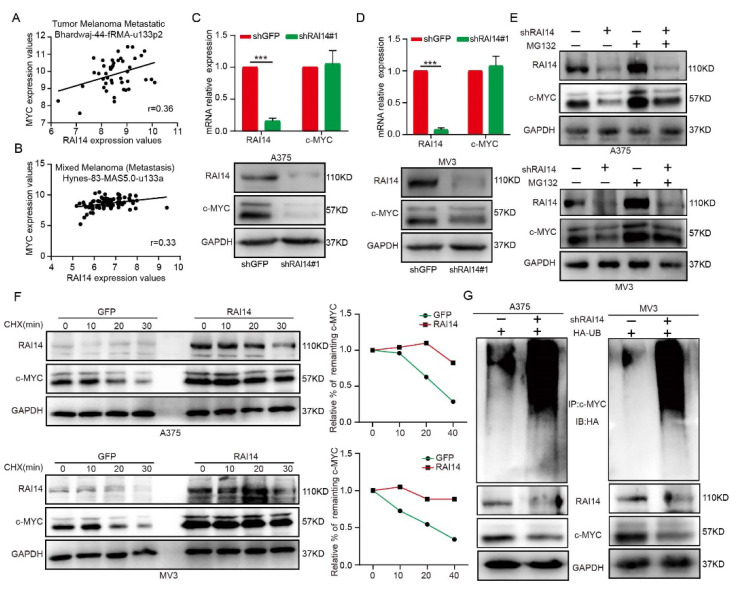
RAI14 regulates the stability of c-MYC by regulating c-MYC ubiquitination. (**A**,**B**) Correlation between RAI14 and c-MYC was analyzed by database analysis. (**C**,**D**) The mRNA and protein of c-MYC were detected by qRT-PCR and Western blot assay. (**E**) Cells were treated with MG132 for 8 h to detect the protein level of c-MYC. (**F**) The effect of RAI14 expression on the turnover rate of c-MYC was explored by treating the RAI14 overexpression group and the control group with CHX with a time gradient (0 min, 10 min, 20 min, 30 min). The gray value was calculated by ImageJ. (**G**) Specific plasmids were transfected into cells, after 40 h, the transfected cells were treated with MG132 for 8 h. The ubiquitinated c-MYC proteins were pulled down with an anti-c-MYC antibody. The data were expressed as mean ± SD. Student’s *t* test was performed to analyze significance. *** *p* < 0.001.

The protein level of c-MYC is upregulated in melanoma cells, and numerous cellular processes are regulated by c-MYC, including cell proliferation, migration, and invasion [19]. Interestingly, the expression of RAI14 was found to correlate with c-MYC signaling in melanoma by analyzing the R2 database. (Figure 3A,B). To further confirm the effect of RAI14 knockdown on the expression of c-MYC in melanoma cells, we performed the Western blot and qPCR assays. Our results indicated that RAI14 can affect the protein level of c-MYC, but not the mRNA level (Figure 3C,D). These results suggested that RAI14 might post-transcriptionally regulate c-MYC. We then treated RAI14 knockdown melanoma cells with MG132 and showed that protein expression of c-MYC was rescued (Figure 3E). We also found that overexpression of RAI14 in melanoma cells reduced the turnover rate of c-MYC (Figure 3F). Moreover, a ubiquitination assay was performed in vitro, and the ubiquitination level of c-MYC was increased in RAI14-knockdown cells (Figure 3G). Taken together, the above results demonstrate that RAI14 may regulate stability of c-MYC by affecting the ubiquitination level of c-MYC.

### 2.5. The Overexpression of c-MYC Significantly Restored Cell Proliferation and Migration of RAI14 Knockdown Melanoma Cells

**Figure 4:**
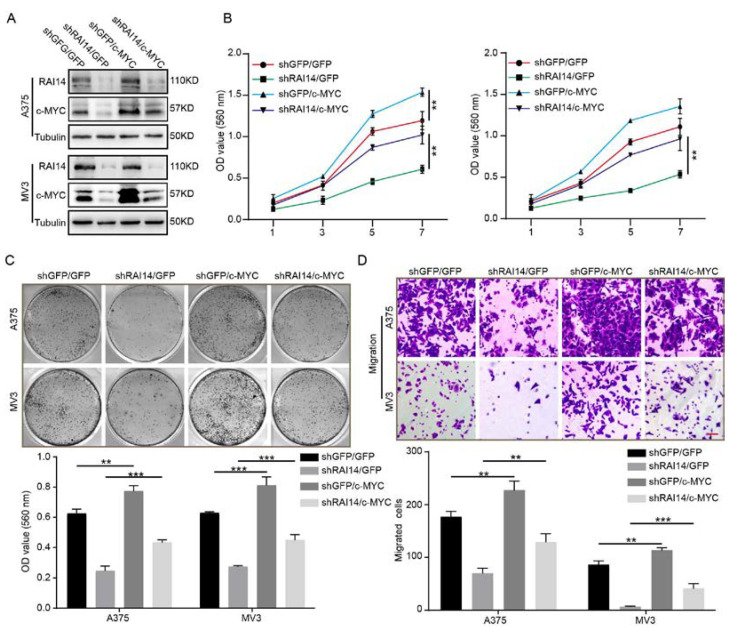
The overexpression of c-MYC significantly restored cell proliferation and migration of RAI14 knockdown melanoma cells. (**A**) The protein levels of RAI14 and c-MYC in the indicated melanoma cells were detected by WB assay. (**B**,**C**) The effect of overexpression of c-MYC on the proliferation of melanoma cells of the RAI14 knockdown group was examined by MTT assay and plate clone assay. (**D**) The effect of c-MYC overexpression on the migration ability of RAI14 knockdown melanoma cells was detected by transwell assay. Scale bar = 50 μm. All data were expressed as mean ± SD. One-way ANOVA was performed to analyze significance. ** *p* < 0.01, *** *p* < 0.001.

To confirm whether RAI14 regulated melanoma progression by targeting c-MYC, we overexpressed c-MYC in RAI14 knockdown melanoma cells (Figure 4A). We found that overexpression of c-MYC significantly restored the inhibitory effect of RAI14 knockdown on cell proliferation by MTT assay and plate clone assay experiments (Figure 4B,C). Furthermore, transwell assay results indicated that overexpression of c-MYC restored cell migration ability of melanoma of the RAI14 knockdown group (Figure 4D). Taken together, these data may suggest that RAI14 regulates melanoma progression by affecting c-MYC expression.

### 2.6. RAI14 Suppressed the Transcription of FBXO32

**Figure 5:**
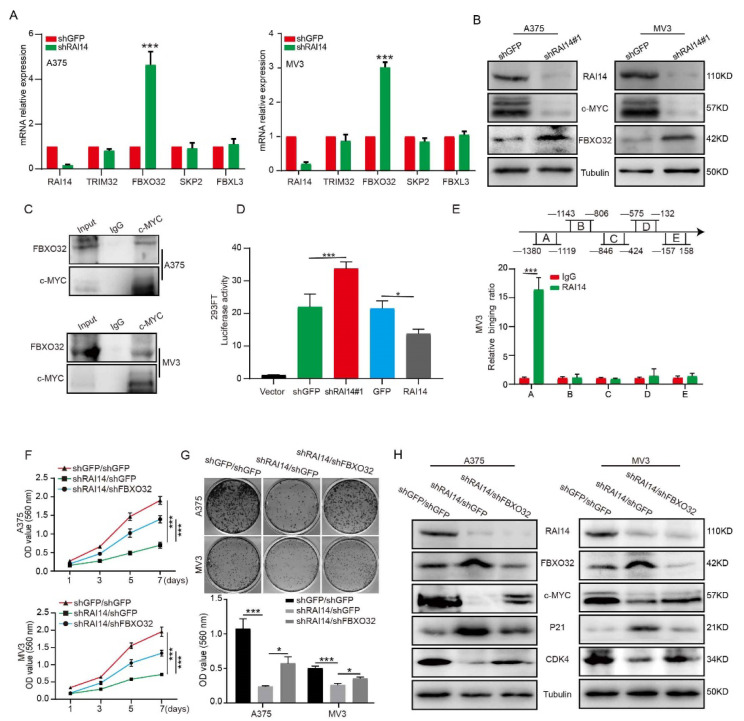
RAI14 suppressed the transcription of FBXO32. (**A**) The mRNA expression level of c-MYC-related E3 ligase was detected through qRT-PCR assay. (**B**) FBXO32 protein expression level was detected by Western blot in RAI14 knockdown group and control group. (**C**) IP was used to detect the protein interaction between c-MYC and FBXO32. (**D**) The transcriptional activity of FBXO32 in control group, RAI14 knockdown group and RAI14 overexpression group were detected. (**E**) ChIP assay was performed by using RAI14 antibodies and IgG was used as the negative control. (**F**,**G**) The cellular viability and proliferation was detected after FBXO32 knockdown. (**H**) The protein levels of RAI14, FBXO32, P21, CDK4 and c-MYC were detected by Western blot analysis. The data were expressed as mean ± SD. Student’s *t*-test and One-way ANOVA were performed to analyze significance. * *p* < 0.05, *** *p* < 0.001.

Some genes with ankyrin repeat domain or coiled-coil domain function as transcription factors or cofactors in cells, such as *CCDC59*, *CC2D1A*, *NKRF*, *RFXANK*, *ANKRD1* and *KANK1*. RAI14 also has the structure of ankyrin repeat domain and coiled-coil domain; therefore, RAI14 may also function as a transcription factor or cofactor in melanoma. Thus, we speculated that RAI14 might regulate c-MYC protein stability by targeting E3 ligase. Therefore, we detected the expression level of c-MYC-related E3 ligase through qRT-PCR assay and found that knockdown RAI14 upregulates the expression of FBXO32 (Figure 5A). Western blot assay demonstrated that FBXO32 expression was increased at the protein levels in RAI14 knockdown groups (Figure 5B). It has been reported that FBXO32 as an E3 ubiquitin ligase, can target and degrade c-MYC. We also validated the results by coimmunoprecipitation, showing that FBXO32 can bind to c-MYC (Figure 5C). To explore whether RAI14 affects the transcription of FBXO32, we performed the dual-luciferase reporter assay. We found that FBXO32 promoter activity was significantly enhanced in RAI14 knockdown cells and reduced in RAI14-overexpressing cells. It indicated that the promoter activity of FBXO32 was inhibited by RAI14 (Figure 5D). To further determine how RAI14 affects FBXO32, we found that RAI14 bound to the A (−1380 to −1119 bp) region of the FBXO32 promoter by ChIP assay (Figure 5E). The above data suggest that RAI14 can affect transcription of FBXO32. To further confirm that RAI14 affects c-MYC ubiquitination through FBXO32, we knocked down the expression of FBXO32 in RAI14 knockdown melanoma cells (Figure S3). We found that knockdown of FBXO32 restored the proliferative capacity of RAI14 knockdown cells by MTT assay and plate clone assay (Figure 5F,G). Western blot assay confirmed that the expressions of c-MYC, CDK4 and P21 were significantly restored after FBXO32 downregulation treatment (Figure 5H). Collectively, the above data suggest that FBXO32 may function as a key downstream factor of RAI14 in melanoma.

### 2.7. RAI14 Knockdown Inhibits Tumor Growth and Improved Prognosis in Mice 

**Figure 6:**
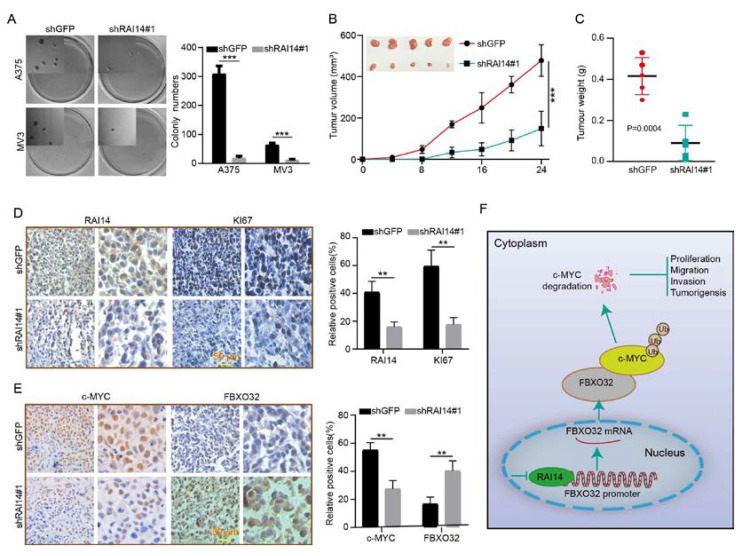
RAI14 knockdown inhibits tumor growth and improved prognosis in mice. (**A**) The effect of knockdown RAI14 on the colony-forming ability of melanoma cells was detected by soft agar assay. (**B**,**C**) Volume and weight of xenograft tumors were counted and analyzed. (**D**,**E**) The expressions of RAI14, Ki67, c-MYC and FBXO32 were detected by immunohistochemical staining. (**F**) A model of the regulatory mechanism of RAI14 in melanoma. The data were expressed as mean ± SD. Student’s *t* test was performed to analyze significance. ** *p* < 0.01, *** *p* < 0.001.

To investigate the effect of RAI14 on tumor growth, we explored the effect of RAI14 on tumor cell self-renewal by soft agar assay. The results showed that knockdown of RAI14 could significantly inhibit the colony-forming ability of melanoma cells (Figure 6A). We also explored the effect of RAI14 on tumor growth in vivo by subcutaneous xenograft assay. The results indicated that the volume and weight of tumors from RAI14 knockdown cells were smaller than the control group (Figure 6B,C). To further validate the effect of RAI14 on tumor growth, we detected Ki67, RAI14, FBXO32 and c-MYC by using immunohistochemistry. The results showed that RAI14 knockdown can significantly reduce the expression of RAI14, Ki67 and c-MYC, whereas the expression of FBXO32 was elevated (Figure 6D,E). It means that knocking down RAI14 inhibits tumor growth in vivo. We draw a model of the regulatory mechanism of RAI14 in melanoma (Figure 6F). Taken together, these data suggest that RAI14 is essential for the growth of melanoma cells. 

## 3. Discussion

Melanoma has the characteristics of high metastasis and high mortality, so the exploration of molecular therapeutic targets is crucial. Recent studies have found that RAI14 is highly expressed in several types of cancer and is associated with cancer prognosis [13,20,21]. However, the biological function of RAI14 in melanoma is unclear, and its molecular mechanism remains to be further explored. Here, we found that high expression of RAI14 is associated with poor prognosis in melanoma patients through the R2 database. We also found that the expression of RAI14 was significantly higher in melanoma than in nevus. Subsequently, we further confirmed the mRNA and protein expression levels of RAI14 in three melanoma cell lines, normal human epidermal melanocytes and immortalized human epidermal cells. We found that the expression of RAI14 was higher in melanoma cell lines than in other cell lines. Moreover, RAI14 was also correlated with the age and sex of the patients through analyzing the database. We further knocked down or restored the expression of RAI14 in melanoma cells by lentiviral interference technology. The results showed that downregulation of RAI14 significantly inhibited the cell proliferation, migration and invasion of melanoma cells, and when the expression of RAI14 was restored, the cell proliferation and migration of melanoma cells were also restored. Therefore, we speculate that *RAI14* may function as an oncogene in melanoma.

Ubiquitination is a significant post-translational modification and is essential for physiological processes such as differentiation, cell survival and immunity [22,23,24]. Ubiquitination of proteins may lead to activation or inactivation of some pathways in cancer [25]. As one of the common oncogenes, *c-MYC* is closely related to the prognosis, occurrence and development of cancer. c-MYC is aberrantly expressed in up to 50% of human cancers [19,26]. Therefore, targeting c-MYC is a potential therapeutic strategy for malignant tumors. In this study, we found that RAI14 can affect the expression of c-MYC in melanoma cells. Treatment of RAI14 knockdown melanoma cells with MG132 partly rescued c-MYC protein expression. We also found that overexpression of RAI14 reduced the turnover of c-MYC by adding CHX. Finally, knockdown of RAI14 can increase the ubiquitination of c-MYC protein by ubiquitination assay. These data suggest that RAI14 affects c-MYC expression through post-translational modification. In addition, restoring the expression of c-MYC could partially restore the proliferation and migration ability of melanoma cells in the RAI14 knockdown group. These data reveal that RAI14 may regulate melanoma growth by affecting c-MYC expression. Subsequently, we speculated that RAI14 might regulate c-MYC protein stability by targeting E3 ligase. Therefore, we detected the expression level of c-MYC-related E3 ligase through qRT-PCR assay and found that knockdown of RAI14 upregulates the expression of FBXO32. The FBXO32 protein expression level was also increased in the RAI14 knockdown group as determined by WB. FBXO32, an E3 ubiquitin ligase, has been shown to target c-MYC for ubiquitin degradation through the proteasome pathway [18]. Furthermore, we found that RAI14 was able to bind to the FBXO32 promoter region to inhibit the expression of FBXO32 by dual-luciferase assay and CHIP assay. However, it is unclear whether RAI14 directly binds to the genome or forms a transcriptional complex and further binds to the genome, which still needs further study.

Current studies have demonstrated that RAI14 is localized in the nucleus and cytoplasm, and found that RAI14 has an evolutionarily conserved nuclear localization signal (P270KKRKAP276) and the nuclear export signal (L352QAKVASLTL361) [27]. RAI14 contains six ankyrin repeats and two coiled-coil domains. We know that ankyrin repeat (AR) domains are the most abundant repeat motif in eukaryotic proteins. It is well-known that AR domains mainly mediate specific protein–protein interactions [28]. Some ankyrin repeat proteins also function as transcription factors or transcription cofactors in cells [29,30,31]. Here, if RAI14 binds directly to the genome, our study indicates the binding range of FBXO32. However, the accurate binding sequence needs to be obtained by the combination of electrophoretic mobility shift assay and ChIP-sequencing assay, while both ankyrin repeats and coiled-coil domains both can mediate protein–protein interactions. Therefore, RAI14 may also form protein complexes with other proteins and then transcriptionally regulate gene expression. 

In summary, our study showed that RAI14 promotes cell proliferation, migration and invasion of melanoma. Furthermore, we found that RAI14 acts as a key factor in regulating c-MYC protein stability by inhibiting the transcriptional activity of FBXO32. These findings provide a new reference for exploring the biological function of RAI14 and reveal that RAI14 may serve as a promising target for melanoma therapy.

## 4. Materials and Methods

### 4.1. Cell Lines, Antibodies, and Reagents

All the immortalized melanoma cell lines (A375, MV3 and SK-MEL-28), immortalized human melanocyte cell line Pig1, human immortalized epidermal cells HaCat and the human embryonic renal cell line 293FT were originally purchased from ATCC (American Type Culture Collection, Rockville, MD, USA). RAI14 (ab137118), RAI14 (ab241499) and FBXO32 (ab168372) were purchased from Abcam (Cambridge, MA, USA); α-Tubulin (2146), c-MYC (#9402S) and HA-Tag (3724S) were purchased from Cell Signaling Technology (Boston, MA, USA). P21 (10355-1-AP), CDK4 (66950-1-Ig), CCND1 (60186-1-Ig), GAPDH (60004-1-Ig), FBXO32 (67172-1-Ig), RAI14 (17507-1-AP) and c-MYC (10828-1-AP) were purchased from Proteintech (Wuhan, China). MG132 (#S2619) was obtained from Selleck Chemicals (Shanghai, China). Cycloheximide (CHX, #C7698) was purchased from Sigma-Aldrich (Shanghai, China). ECL reagents was obtained from Beyotime (#P0018, Shanghai, China). RIPA lysis buffer was purchased from Beyotime (Shanghai, China). Certified Fetal Bovine Serum (FBS) was purchased from VivaCell (VivaCell, Shanghai, China). Propidium iodide (PI) was purchased from BD Biosciences. Dulbecco’s modified Eagle’s medium (DMEM) was purchased from BD Becton, Dickinson and Company. 

### 4.2. Transfection and Infection

shRAI14, shFBXO32 and a control shGFP sequences were inserted into the pLKO.1 vector. shRNAs sequences were obtained from Gene Pharma Co., Ltd. (Shanghai, China) The sequences are as follows: (shRAI14#1:5′-CCGGGCAGACCTAAACCTTGTAGATCTCGAGATCTACAAGGTTTAGGTCTGCTTTTTG-3′), (shRAI14#2:5′-CCGGCCGCTGCCATTGTTCTCATTCCTCGAGGAATGAGAACAATGGCAGCGGTTTTTG-3′) and (ShFBXO32: 5′-CCGGGCAACAAGGAGGTATACAATACTCGAGTATTGTATACCTCCTTGTTGCTTTTTG-3′). The UB plasmid that contained an HA tag was purchased from Addgene (Beijing, China). RAI14 full-length cDNA recombinant plasmid containing PCDH-CMV-MCS-EF1-Hygro vector was purchased from Youbao Company (Changsha, China). For transfection and infection experiments, the target plasmid and packaging plasmid were transfected into 293FT cells by using the transfection reagent Lipofectamine 2000 (Invitrogen, Carlsbad, CA, USA). The lentivirus was collected after 48 h, and tumor cells were then infected with the lentivirus twice for 24 h each. Cells were selected with puromycin for 48 h after medium change. Surviving cells were used for subsequent experiments. 

### 4.3. Quantitative Real-Time RT-PCR Assay 

RNA was extracted using Trizol (Invitrogen) and 2 ug RNA was reverse-transcribed into DNA. Real-time quantitative PCR was performed using SYBER Green PCR Master mix (Takara) and LightCycler96 Real-Time PCR System (Roche, Indianapolis, IN, USA). Using GAPDH as an internal reference, the relative expression levels were calculated by the 2^−∆∆Ct^ method. Total RNA extraction, reverse transcription and real-time PCR were reference to a report [32]. Related primers are shown in Supplementary Table S1.

### 4.4. Cell Proliferation Assay (MTT Assay)

Cells were grown in 96-well plates at 1000 cells per well. The medium was changed every two days. MTT was added, the medium was removed after four hours and OD was measured after addition of DMSO. OD values were measured on days 1, 3, 5 and 7, respectively. Growth curves were drawn based on OD values.

### 4.5. BrdU Assay

Bromodeoxyuridine (BrdU) assay can detect the rate of cell DNA replication. We added 10,000 cells to a 24-well plate and cultured them for 2 days. BrdU (Sigma, Shanghai, China) was then added and incubated for 40 min. Subsequently, the cells were washed 3 times with PBS and fixed with 500 µL 4% PFA for at least 20 min. After treatment with 2 M hydrochloric acid and 0.1% Triton-100 for 30 min, cells were blocked with 5% goat serum for 1 h at room temperature. Then, BrdU antibody was added and incubated overnight at 4 °C. After washing the cells with PBS three times, the Alexa flour 594 secondary antibody was incubated for 1 h. DAPI was added to stain for 20 min. At least three areas to photograph under the microscope were selected. 

### 4.6. Flow Cytometry

Cells were harvested by trypsinization and fixed with 75% ethanol at −20 °C for 48 h. After washing the cells three times with PBS, PI (propidium iodide) and RNase were added and incubated at 37 °C for 30 min and were analyzed by flow cytometer (BD c6, Piscataway, NJ, USA).

### 4.7. Plate Clone Formation and Soft Agar Assays 

Plate clones were used to measure cell proliferation. Briefly, cells were seeded in 6-well plates at 1000 per well and cultured for 7 days. A total of 2 mL of 4% paraformaldehyde was added to fix the cells. Then, they were stained with 0.5% crystal violet for 30 min and imaged with an Epson Perfection PhotoV700 scanner (Epson (China) Co., Ltd, Beijing, China). Ethanol was added to dissolve crystal violet and the OD value was measured.

Soft agar assay measures the self-renewal and colony-forming capacity of cells. A total of 2× DMEM and 1.2% agarose were mixed in a 1:1 ratio in a tube and added to a 6-well plate. 1000 cells were mixed in 1× DMEM medium, then mixed with 2× DMEM and 1.2% agarose at a ratio of 2:1:1 and added to the solidified lower gel as the upper gel. After culturing for 18–25 days, MTT was added for staining at 37 °C for 30 min, and the images were scanned with a scanner and the colonies were counted.

### 4.8. Transwell Assay

Cell migration/invasion capacity was determined using Boyden chambers (8-μm pore size, Corning, New York, NY, USA). A total of 500 mL medium was added to a 24-well plate, then 2 × 10^5^ cells were mixed with 200 μL serum-free medium and added to transwells. After culturing for 6–24 h, 500 μL of 4% paraformaldehyde was added to the 24-well plate and the chamber was placed in PFA for 20 min. The chambers were then stained in 0.1% crystal violet. Finally, the cells in the Boyden chamber were wiped off with cotton wool. The stained cells were examined in the microscope and counted by Image J 2.1.0 (NIH, Bethesda, MD, USA). The procedure for the invasion assay was similar to the migration assay, except that the transwell membranes were precoated with Matrigel (R&D Systems, New York, NY, USA). 

### 4.9. Western Blot Analysis

Proteins were extracted from cells by RIPA buffer supplemented with protease inhibitors. The protein was separated on the gels by sodium sulfate polyacrylamide gel electrophoresis. The protein was then transferred to PVDF membrane, blocked with 5% milk for 2 h and incubated with primary antibody overnight at 4 °C. The primary antibody was recovered and the PVDF membrane was washed with TBST and incubated with an appropriate horseradish peroxidase (HRP)-conjugated secondary antibody for 2 h. Membranes were then visualized using an enhanced chemiluminescence detection kit. Finally, the membrane was exposed in the gel imaging system (Qinxiang, Shanghai, China).

### 4.10. Ubiquitination and Turnover Assay

For the ubiquitination assay, shGFP, shRAI4 and HA plasmids were transfected into A375 and MV3 cells by Lipofectamine 2000 (Invitrogen, Carlsbad, CA, USA). After 40 h, MG132 was added to the cells and cultured for 8 h. Cells were harvested and extracted proteins were lysed with IP lysis buffer. Primary antibody and protein A/G magnetic beads were added and incubated overnight at 4 °C. The beads were washed 5 times with PBS, denatured at 100 °C and detected by Western blotting.

For the turnover assay, the specific plasmid was transfected into melanoma cells and screened with puromycin and hygromycin B for 48 h. After the transfected gene was stably expressed, CHX was added to incubate and the cells were collected according to the time gradient.

### 4.11. Dual-Luciferase Reporter Assay 

The FBXO32 promoter recombined into the PGL3 vector, PGL3-TK and specific plasmids were cotransfected into 293FT. After 48 h, follow-up operations and analysis of experimental results were performed according to the instructions of the dual-luciferase kit (Promega, Beijing, China). 

### 4.12. Chromatin Immunoprecipitation

The CHIP Assay was performed according to the instructions of the ChIP kit (Beyotime: Shanghai, China). Briefly, formaldehyde was added to cells for cross-link proteins/DNA. Glycine solution was added and mixed gently, and the mixture was incubated at room temperature for 5 min. Cells were lysed using SDS lysis buffer and sonicated. A total of 10 μg antibody or IgG antibody was added and incubated at 4 °C for 2 h. Protein G beads were added and incubated overnight at 4 °C. The DNA was then purified by DNA Gel Extraction Kit (Beyotime, Shanghai, China) and finally detected by PCR. Related primers are shown in Supplementary Table S2.

### 4.13. Xenograft Assay 

Four-week-old female NOD/SCID mice were purchased and housed in SPF room. The mice were randomly divided into two groups. A melanoma cell line (A375) stably transfected with shGFP and shARI14#1 (1 × 10^5^ cells) was injected into subcutaneous tissue of mice (5 in each group). The injection site of the mice was sterilized with 75% medical alcohol. Tumor diameters were measured every 4 days. The tumor volume was calculated as follows: V = (length × width^2^)/2. Randomization and single blinding were used for measurement. After 24 days, the pain of the mice was relieved using an isoflurane anesthesia system, then the mice were sacrificed by cervical dislocation and the tumors were harvested. Tumors were collected and photographed and weighed. The bodies of mice were frozen at −20 °C and then transferred to Laibite Biotech Inc. (Chongqing, China) for incineration. All studies were approved by the Animal Care and Use Committee of Southwest University and carried out in conformity to the Guide for the Care and Use of Laboratory Animals (Ministry of Science and Technology of China, 2006). 

### 4.14. Immunohistochemistry

Immunohistochemistry staining was reference to a report [33]. Briefly, paraffin-embedded tumors were cut into 5 μm-thick sections, which were then deparaffinized and hydrated. Then, after antigen retrieval, endogenous peroxidase was blocked by treatment with 0.3% hydrogen peroxide for 10 min and blocked with 4% goat serum for 1 h. The antibody was dropped onto the sliced tissue and incubated overnight at 4 °C. After washing the sections, secondary antibody was added and incubated for 40 min. 3,3′-diaminobenzidine was added dropwise for color development. Sections were dehydrated and mounted after hematoxylin staining. Images were taken with an Olympus microscope. (Olympus CKX41; Olympus Corp., Tokyo, Japan).

### 4.15. Patient Data Analysis

Patient data and gene expression data were obtained from the R2 database (https://hgserver1.amc.nl/cgi-bin/r2/main.cgi) (accessed on 1 July 2022).

### 4.16. Statistical Analysis

Graphpad prism software was used to analyze the data. Two-tailed Student’s *t* test was performed for two groups and one-way ANOVA was used for three or more groups to calculate significance at the 95% confidence level. * *p* < 0.05, ** *p* < 0.01, *** *p* < 0.001 indicate different degrees of statistical significance, as shown.

## Data Availability

The data presented in this study are available on request from the corresponding author.

## Supplementary Materials

The following supporting information can be downloaded at: https://www.mdpi.com/article/10.3390/ijms231912036/s1.
