# Supplementary material for: RAI14 Promotes Melanoma Progression by Regulating the FBXO32/c-MYC Pathway
Source: Int J Mol Sci. 2022 Oct 10;23(19):12036. doi: 10.3390/ijms231912036 (PMC9569902; doi:10.3390/ijms231912036)
Supplement: Supplementary file 1 [file ijms-23-12036-s001.zip › ijms-1900529-supplementary.pdf]

Supplementary

Table S1. RT-PCR primers.

RAI14-F	AGCCCAAGATACTACCGGACA
RAI14-R	CGCTGCATAATGTAAAGCTGTTT
FBXO32-F:	ATGAGAAGAGCGGCAGTTTC
FBXO32-R:	TTCTTTTGGGCGATGCCACT
c-MYC-F	GTCAAGAGGCGAACACACAAC
c-MYC-R	TTGGACGGACAGGATGTATGC

Table S2.

RT-PCR primers

FBXO32-1-261-F	ACAGAGACCAGACCCAACACTGCC
FBXO32-1-261-R	ACTGACTCGGTGTAATCTTTAAGGC
FBXO32-237-574-F	CCTTAAAGATTACACCGAGTCAGT
FBXO32-237-574-R	TCTGCAGTGGGAACTCCATGAGAGC
FBXO32-556-956-F	GAACAAGACAGACCAGCTCTCATG
FBXO32-556-956-R	CTGGGGCCGGCACCGCGCCCGCCGG
FBXO32-934-1248-F	GGTTAGTGACAGCTAAGGGG
FBXO32-934-1248-R	TGGAAACTTGAAGCGGTGCT
FBXO32-1227-1538-F	AGCACCGCTTCAAGTTTCCAC
FBXO32-1227-1538-R	GGGGTGCAGGGGCCCGCGA

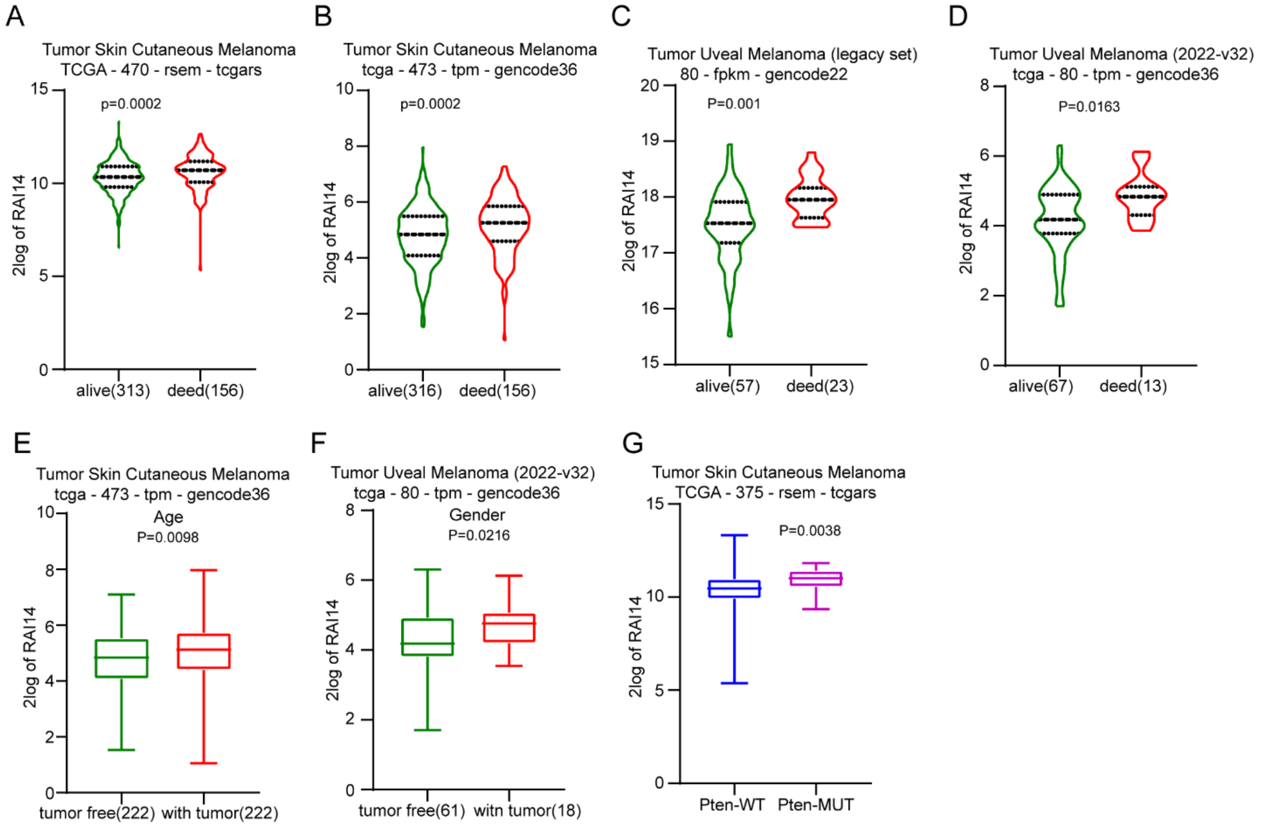

Supplementary Figure S1 High expression of RAI14 is associated with poor prognosis of Melanoma

A, B, C, D Analyses of RAI14 expression in living patients and in dead patients from four different databases. **E, F** The expression level of RAI14 in the tissues of tumor with-melanoma patients and tumor free (surgically removed) -melanoma patients. **G** The expression of RAI14 in Pten wildtype status and Pten mutation status. The data were expressed as mean ± SD. Student's t test was performed to analyzed significance.

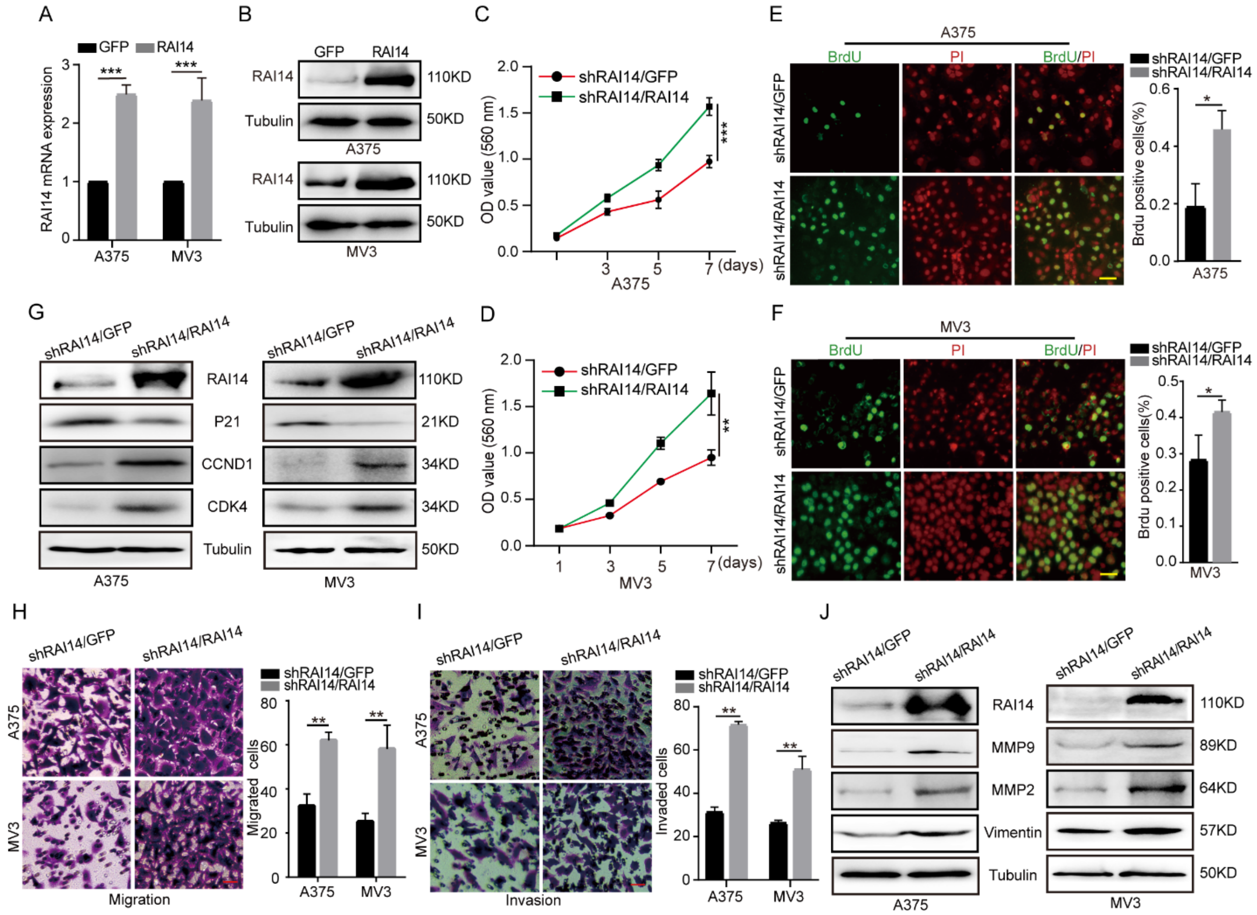

Supplementary Figure S2 RAI14 recovery restored the cell proliferation, migration, and invasion of RAI14-knockdown Melanoma cells

A, B Western Blot and qRT-PCR assay were performed to prove the recovery of RAI14. **C, D** MTT assays were performed to examine the effect of RAI14 overexpression on the cell viability and proliferation ability of RAI14-knockdown cells. **E, F** BrdU assay was used to detect the DNA synthesis ability after RAI14 restoration in RAI14 knockdown cells. **G** Western blot assay was executed to detect the expression of G1 cell cycle regulatory proteins after RAI14 restoration in RAI14 knockdown cells. Scale bar = 50 μm. **H, I** Transwell assays were used to detect the effect of RAI14 overexpression on the migration and invasion of RAI14-knockdown and control cells. **J** Western blot assays were used to detect the effect of RAI14 overexpression on the EMT-related proteins of RAI14-knockdown and control cells. The data were expressed as mean ± SD. Student's t test was performed to analyzed significance. *P < 0.05, **P < 0.01, ***P < 0.001.

S3

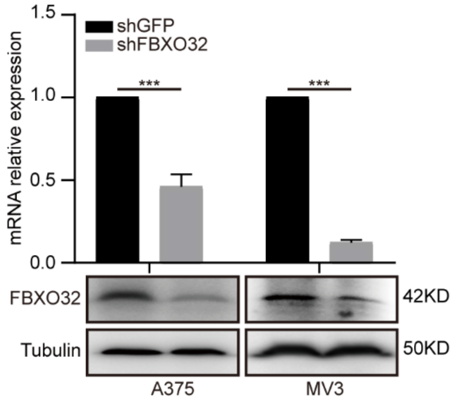

Supplementary Figure S3 Western Blot and qRT-PCR assay were performed to detect the effect of the FBXO32 knockdown. *** $p < 0.001$.
